# Undesirable Difficulty Effects in the Learning of High-Element Interactivity Materials

**DOI:** 10.3389/fpsyg.2018.01483

**Published:** 2018-08-13

**Authors:** Ouhao Chen, Juan C. Castro-Alonso, Fred Paas, John Sweller

**Affiliations:** ^1^National Institute of Education, Nanyang Technological University, Singapore, Singapore; ^2^Center for Advanced Research in Education (CIAE), Universidad de Chile, Santiago, Chile; ^3^Department of Psychology, Education, and Child Studies, Erasmus University Rotterdam, Rotterdam, Netherlands; ^4^School of Education/Early Start, University of Wollongong, Wollongong, NSW, Australia; ^5^School of Education, University of New South Wales, Sydney, NSW, Australia

**Keywords:** cognitive load theory, human cognitive architecture, desirable difficulties, element interactivity, testing and generation effects

## Abstract

According to the concept of desirable difficulties, introducing difficulties in learning may sacrifice short-term performance in order to benefit long-term retention of learning. We describe three types of desirable difficulty effects: testing, generation, and varied conditions of practice. The empirical literature indicates that desirable difficulty effects are not always obtained and we suggest that cognitive load theory may be used to explain many of these contradictory results. Many failures to obtain desirable difficulty effects may occur under conditions where working memory is already stressed due to the use of high element interactivity information. Under such conditions, the introduction of additional difficulties may be undesirable rather than desirable. Empirical evidence from diverse experiments is used to support this hypothesis.

## Introduction

There is considerable data available indicating that introducing difficulties during instruction may slow down the acquisition rate of learning, but facilitate long-term retention and transfer (Bjork and Linn, 2006; Roediger and Karpicke, 2006; Rohrer and Taylor, 2007; Soderstrom and Bjork, 2015). These instructional difficulties are known as *desirable difficulties*. In this review paper, we will discuss theoretical and empirical work in the context of cognitive load theory to argue that the effectiveness of desirable difficulties in learning may be moderated by the working memory load imposed by the instructional material. Working memory load is determined by available working memory capacity and levels of element interactivity defined by a combination of the complexity of the information and levels of learners’ expertise. In this paper, we focus on the testing and generation effects that are desirable difficulties that have been moderated by varying levels of element interactivity.

## Desirable Difficulties

The framework of desirable difficulties is based on the assumption that including some difficulties in students’ learning may lead to long-term retention and transfer of knowledge (Bjork, 1994). Such difficulties may include: *testing* – including retrieval practice of taught materials compared to re-visiting them; *generation* – self-generating answers compared to studying presented answers; and *varied conditions of practice* – learning in multiple environments compared to a single environment.

### Testing Effect

Considerable research has shown the pedagogical advantages of testing. McDaniel et al. (2007) investigated the testing effect with a college course. A group of students took quizzes before taking a final multiple-choice test, while, another group of students was presented with target materials for reading instead of the quizzes prior to the final multiple-choice test. The multiple-choice test results favored using quizzes over additional reading. In medical education, Kromann et al. (2009) used a controlled, randomized intervention study to compare students who studied and practiced followed by tests, to students who only studied and practiced. After 2 weeks, the results showed a testing effect, indicating that testing enhanced skills compared to spending an equal amount of time on practicing as a final activity. Many other studies also have shown the testing effect (e.g., Chan and McDermott, 2007; Agarwal et al., 2008; Johnson and Mayer, 2009). In this paper (here and in the Section “Future directions for research”), all testing is defined as problem solving whether the test solely requires retrieval of information from long-term memory as occurs when the test-taker is an expert in the area or when testing involves a combination of retrieval from long-term memory and the generation of novel responses as occurs with novices.

The advantage of testing can be explained by both storage and retrieval processes (Wheeler et al., 2003). Storage occurs during learning and retrieval is induced by testing. Meta-analyses of the testing effect have been conducted, showing the robustness of this effect with overall effect sizes of 0.50 (Rowland, 2014) and 0.56 (Schwieren et al., 2017). The nature of the learning materials (e.g., the type of stimuli) may constitute a moderator (Rowland, 2014; Adesope et al., 2017; Pan and Rickard, 2018).

However, there might be additional moderators, as research has also shown failures in obtaining testing effects in acquiring problem-solving skills. For example, several studies (van Gog and Kester, 2012; Leahy et al., 2015; van Gog et al., 2015; Hanham et al., 2017) using cognitive load theory compared a worked example only condition (study-study) to a worked example-problem solving condition (study-testing), but over many experiments obtained a mix of results favoring testing, reverse testing where additional studying was superior to testing, or no difference between conditions. Reasons for these contradictory results are provided below in our discussion of cognitive load theory.

### Generation Effect

The generation effect describes the finding that generating one’s own answers rather than studying the answers of others may have long-term advantages for learning (Slamecka and Graf, 1978). The generation effect differs from the testing effect in the sequence of study and testing. The testing effect compares a study-testing with a study–study sequence while the generation effect compares a generating condition with a study or presentation condition. Traditionally, research into the generation effect has used word pairs. Glisky and Rabinowitz (1985) applied single words with missing letters (e.g., ALC-H-L) in their experiments. One group of students generated the missing letters to complete the word compared to another group of students who were presented the missing letters. The experiment may suggest that the access to semantic memory improves performance on an episodic memory test, e.g., when using words with missing letters. Also, Anderson et al. (1971) applied incomplete sentences as contexts in which the to-be-generated target was a highly probable completion, such as “The doctor looked at the time on his (watch)”.

A meta-analysis of the generation effect by Bertsch et al. (2007) showed that the effect was robust (overall effect size of 0.40 across 86 studies). The analysis also showed that the difficulty of the task was a significant moderator, and simple tasks such as simple math calculations and word fragment completions showed larger effects. The generation effect has had multiple explanations, such as generation activates or strengthens both response-specific features and the relation between a stimulus and a response (Hirshman and Bjork, 1988).

However, similar to the testing effect, some research studies also have demonstrated a failure in obtaining the generation effect. The studies of McElroy and Slamecka (1982) and Lutz et al. (2003) suggested that materials used in the generation effect may need to be semantically meaningful. They found no generation effect for non-words, even if these non-words were readable. Similar results were found when the materials were meaningless letter bigrams (e.g., E C), non-unitized 2-digit numbers (e.g., 2, 8), and unfamiliar compounds (e.g., cheese ketchup) (Gardiner and Hampton, 1985). A failure to obtain the generation effect under some specific conditions also has been demonstrated in recent research studies (Chen et al., 2015, 2016a,b) that are discussed in more detail below.

### Varied Conditions of Practice

Studying in a constant and predictable condition may facilitate immediate retrieval of learning materials, but show no advantages for knowledge transfer and long-term retention (Bjork and Bjork, 2011). In contrast, varying the conditions of practice may impair performance during acquisition but may enhance long-term performance. Smith et al. (1978) tested environmental context effects with recall and recognition of word lists. They found an advantage of varying context practice over constant context practice on a free recall test, but a reversed effect was found on a recognition test and a recall test of categories and words from the same category. Similar results have been found with problem solving tasks (e.g., Reder et al., 1986). The theory of encoding variability is often used to explain the advantage of varied conditions of practice (Smith and Handy, 2014).

The difficulty added to learning tasks by varying the conditions of practice may not always be effective. Paas and van Merriënboer (1994) compared both low- and high-variability conditions with either conventional problem solving or with worked example studying. For both conventional and worked example conditions, the low variability condition varied the values only, but the high variability condition varied both the values and format of questions. The results indicated that adding variability to the format of questions was effective in combination with worked examples, but ineffective in combination with conventional problem solving.

### Summary of the Desirable Difficulties Research

As can be seen, there is substantial evidence for a variety of desirable difficulty effects. Nevertheless, there also is evidence of failure to find expected effects and, as will be indicated below, considerable evidence for reverse desirable (or undesirable) difficulty effects. As we will argue, the reverse desirable (or undesirable) difficulty effects, indicated below, are caused by the element interactivity of the learning materials. McDaniel and Butler (2011) also discussed how relations among difficulties, the nature of the learning materials, and the properties of the learners may affect desirable difficulties. However, there is not a concept in the desirable difficulty framework that could be used to measure the difficulty of the learning materials. We suggest that cognitive load theory and the concept of element interactivity can be used as a possible option for measuring the difficulty and complexity of learning materials and, in the process, explain some of the apparent contradictions in the research base.

## Cognitive Load Theory and Human Cognitive Architecture

Human cognitive architecture provides a base for cognitive load theory, which is an instructional theory. Instructional design and human cognitive architecture are inseparably intertwined (Sweller et al., 1998, 2011). Knowing how students learn and solve problems informs us how we should organize their learning environments. Five principles indicate the pedagogical aspects that flow from human cognitive architecture. These five principles also describe the manner in which evolution by natural selection processes information (Sweller and Sweller, 2006).

### The Information Store Principle

In order to function in a complex, natural environment, humans must be able to store large amounts of information. That information is stored in long-term memory. The goal of instruction is to increase knowledge stored in long-term memory. Based on the information store principle, difficulties will be desirable if they increase the amount and speed that information can be stored in long-term memory. Difficulties will be undesirable if they interfere with the storage of information in long-term memory.

### The Borrowing and Reorganizing Principle

Given the enormous amounts of information that must be acquired to be stored in long-term memory, efficient procedures are required to obtain that information in a timely fashion. Humans have evolved to “borrow” instructionally relevant information from other people. We imitate others, listen to what they say, and read what they write. The information is re-organized before being transferred to long-term memory to cohere with currently stored information.

### Randomness As Genesis Principle

Sometimes, required information is not available from others and so must be generated. We generate novel information by using a random generate and test procedure during problem solving. Problem solving moves can be randomly generated and tested for effectiveness with successful moves retained in long-term memory and unsuccessful ones discarded.

### Narrow Limits of Change Principle

Very large, rapid changes to long-term memory can be dysfunctional and using a random generate and test procedure when dealing with more than a few elements of novel information can result in combinatorial explosions that also can render the procedure dysfunctional. In order to avoid those problems, limits on the amount of novel information that can be processed are required. Those limits are provided by the limitations of working memory, which has a very limited capacity (Miller, 1956; Cowan, 2001) and duration (Peterson and Peterson, 1959). Cognitive load theory assumes that these limits only apply when dealing with novel information. Any difficulties we add to information will be undesirable if there is not enough working memory capacity to deal with them. If they are within working memory limits, then they may potentially have positive (desirable) effects.

### Environmental Organizing and Linking Principle

Once information has been structured and stored in long-term memory, it can be retrieved by working memory to generate action that is appropriate for a given environment without the limitations associated with processing novel information. This principle provides the transformational character of education. We are able to engage in learned activities that otherwise we could not possibly carry out.

The environmental organizing and linking principle is critical to human cognitive architecture and leads directly to the concept of element interactivity below. Once information is stored in long-term memory, it alters the characteristics of working memory and so may alter the desirability or undesirability of additional difficulties. Because the information stored in long-term memory is chunked, the amount of information that can be processed by working memory is reduced. Hence, a difficulty that may be desirable for a more knowledgeable learner may be undesirable for a less knowledgeable learner. The difficulty and complexity of information will depend not just on the characteristics of the information but on the knowledge of the person processing the information. Element interactivity considers both of these factors simultaneously.

### Instructional Consequences of Human Cognitive Architecture

Cognitive load theory uses this cognitive architecture to devise instructional effects, such as the worked example effect. For novices learning from worked examples, cognitive load is relatively low and overloading of working memory capacity is avoided through the *borrowing and reorganizing principle*. In contrast, when learning from problem solving, novices’ cognitive load is relatively high and their working memory capacity is easily overloaded through the *randomness as genesis principle*, which is a result of the means-ends strategy that they use to solve problems. Note that testing and generation share the same mechanism of problem solving. From that perspective, it can be argued that the comparison between studying worked examples only and studying worked examples followed by problem solving is analogous to the comparison between repeated reading and reading followed by recalling information. Importantly for the purposes of the current paper, some of the cognitive load effects such as the worked example effect directly contradict the concept of desirable difficulties. Nevertheless, by use of the concept of element interactivity, which is central to cognitive load theory, some of the conditions under which desirable difficulty phenomena should and should not be manifested can be predicted.

## Element Interactivity

Element interactivity can be determined by estimating the number of interacting elements in learning materials (Sweller and Chandler, 1994; Tindall-Ford et al., 1997; Sweller, 2010). Interactive elements are defined as elements that must be processed simultaneously in working memory as they are logically related (Sweller et al., 2011). An element which should be processed in working memory can be a symbol or a concept, and it is characteristically a schema. Element interactivity is not only determined by the characteristics of the learning materials, but also determined by the levels of learners’ expertise (Chen et al., 2017).

### Element Interactivity Determines Types of Cognitive Load

Element interactivity determines the three types of cognitive load: intrinsic load, extraneous load and germane load (Sweller et al., 2011). Intrinsic load reflects the nature of learning materials and is positively related to the number of interactive elements of learning materials. Extraneous load, imposed by suboptimal instructional design, depends on the number of interactive elements that are present not because of the nature of the information but because of the way the information is presented. Germane load refers to the actual working memory resources allocated to deal with intrinsic cognitive load. It relies on the number of interactive elements that are intrinsic to the learning materials. Therefore, learning materials that include more intrinsic interactive elements impose a greater cognitive load compared to materials with fewer intrinsic interactive elements.

### Element Interactivity and the Characteristics of the Learning Material

The nature of the learning material influences the level of element interactivity which determines the level of cognitive load imposed on working memory. For example, learning the translation of words from one language to another provides an example of material low in element interactivity and so imposing a low level of cognitive load. When a student memorizes the word “cat” in a foreign language, which is 1 new element that needs to be learned, there is no need to refer to the translation of any other words. Therefore, the number of interactive elements should be 1 for memorizing a list of individual vocabulary words. In contrast, if a student is required to solve an equation, such as 2*x* + 5 = 3 for *x*, there may be over a dozen or more interconnected elements (e.g., the algebraic elements such as 2, *x*, +, along with the relations between them) that must be simultaneously processed in working memory. Therefore, this type of material is high in element interactivity resulting in a high level of cognitive load.

It needs to be noted that element interactivity is related to but not equivalent to difficulty. Learning the translation of a long list of words may be far more difficult than learning to solve an algebraic equation but imposes a far lower working memory load. Element interactivity refers to working memory load, not difficulty.

### Element Interactivity and Levels of Expertise

Levels of learners’ expertise also affect levels of element interactivity. When solving the above equation, for a novice, the number of interactive elements may be over 12, which will exceed working memory capacity, whereas, for an expert, the number of interactive elements may be reduced to 1. An expert who can retrieve knowledge of the equation and its solution as a single entity from long-term memory using the environmental organizing and linking principle, treats the equation and its solution as a single element. Therefore, material that is high in element interactivity for a novice in the area will be low in element interactivity for an expert. Notwithstanding, if the number of elements remains constant but interactivity alters, difficulty also will alter. For this reason, element interactivity should be an essential component of discussions of desirable difficulties. By doing so, a clearer picture of desirable difficulties may be drawn.

## Element Interactivity May Moderate the Effectiveness of Desirable Difficulties

Evidence collected thus far from studies based on cognitive load theory indicates that two desirable difficulties, the testing and the generation effects, are effective for low but ineffective for high element interactivity information. For another desirable difficulty, varied conditions of practice, a similar hypothesis can be made. That evidence is discussed next.

### Element Interactivity Moderates Testing Effects

In a review, van Gog and Sweller (2015) indicated that evidence for the testing effect was more likely to be obtained using less rather than more complex information. That evidence dates back to the earliest demonstrations of the effect early last century. There also is more recent evidence. Leahy et al. (2015) investigated the testing effect by teaching primary students to read a bus timetable. Students were randomly assigned to a worked example-problem solving condition (i.e., learning followed by testing) and a worked example followed by another worked example condition (i.e., learning followed by re-learning). Experiments 1 and 2 consistently showed a reversed testing effect, indicating that students in the worked examples only group achieved higher results than those in the worked example-problem solving group. In Experiment 3, a 1-week delayed test was used to investigate the testing effect using similar materials but still, no testing effect was found. The possible reason used to explain these results was that learning to use a bus timetable for primary school students was a high element interactivity task. Hanham et al. (2017) investigated the testing effect with materials that were either low or high in element interactivity. In multiple experiments, two groups were compared: a worked examples only group constituting study only and a worked example-problem solving group where problem solving constituted testing. Experiments using low element interactivity information yielded a testing effect while experiments using high element interactivity information either indicated no effect or a reverse testing effect. van Gog and Kester (2012) investigated the testing effect when solving electrical circuit troubleshooting problems. The students learned either with worked examples only or with worked example-problem solving pairs. On an immediate test, there were no differences between the groups. However, a delayed test showed that the worked example only condition outperformed the worked example-problem solving group, yielding a reverse testing effect. Several experiments by van Gog et al. (2015) using similar, high element interactivity, problem-solving materials provided no evidence of a testing effect.

These results can be explained from a cognitive load theory perspective. To understand and learn high element interactivity information, learners are likely to require multiple passes through the material. After a single pass, they are likely to have only partially understood and learned the material and so require additional practice before the information is consolidated in long-term memory, resulting in superior performance by students presented opportunities for that additional practice. In contrast, low element interactivity information may be understood and learned after a single pass. Additional passes may be redundant and presenting learners with redundant information interferes with learning (see Sweller et al., 2011 for a summary of the redundancy effect). Accordingly, providing learners with a test rather than additional, redundant study time is beneficial.

### Element Interactivity Moderates Generation Effects

Again, we need to notice that the generation effect differs from the testing effect in the sequence of study and testing. The testing effect compares a study-testing with a study–study sequence, while the generation effect compares a sequence of worked-example study and problem solving with a sequence consisting only of worked-example study. Chen et al. (2015) investigated the effects of differing levels of element interactivity on both the generation and the worked example effects. The two effects are contradictory. The generation effect suggests that having learners generate responses rather than studying information is beneficial while the worked example effect suggests that asking learners to study appropriate information is beneficial compared to generating it. Chen et al. (2015) found that, for novices, low element interactivity material such as learning geometric formulae produced the generation effect while high element interactivity information such as learning to use the formulae to solve geometry problems produced a worked example effect. When testing more expert learners for whom both learning the formulae and learning to solve problems was low in element interactivity, a generation effect was found. Additional experiments were conducted by Chen et al. (2016a,b) using similar experimental designs. The results again confirmed that the generation effect was obtained only for materials low in element interactivity and the worked example effect was obtained using high element interactivity information. In addition, Chen et al. (2016a) found these effects on delayed tests.

Again, these results can be explained by cognitive load theory using a similar explanation to the testing effect results. High element interactivity information imposes a high working memory load that can be reduced by using worked examples rather than problem solving. Additional difficulties such as generating a solution can be considered undesirable difficulties rather than desirable difficulties. In contrast, low element interactivity information does not require worked examples. Instead, worked examples are redundant and undesirable. With simple materials, generating a response can be considered a desirable difficulty rather than an undesirable difficulty, resulting in a generation effect.

### Element Interactivity Moderates Varied Conditions of Practice

Paas and van Merriënboer (1994) used the cognitive load theory framework to investigate the effects of variability of practice when novices studied worked examples or solved problems. They compared a low-variability practice condition, in which the same problem format was used with different values, with a high-variability learning condition, in which both values and problem format were varied. In these conditions, higher variability implied higher element interactivity, as there were more elements to manage simultaneously in working memory. Because problem solving imposes a higher working memory load on novices than studying worked examples, it was hypothesized that higher variability would be effective in combination with worked-example practice and ineffective in combination with conventional problem solving practice. Accordingly, for worked examples, the introduction of variability constitutes a desirable difficulty. The hypothesis was confirmed by the results. When studying worked examples, increased variability resulted in increased learning.

We can predict that in line with the argument and results obtained from the generation and testing effects, an increase in element interactivity should eliminate or reverse the variability effect with low rather than high variability leading to improved performance. Additional difficulties that increase element interactivity may not be desirable if element interactivity is already so high that it exceeds working memory capacity. In contrast, increasing element interactivity when it is low may be beneficial provided that the increase in element interactivity does not exceed working memory capacity. If element interactivity is already high, adding to it by introducing variability may result in worked memory capacity being exceeded with deleterious effects on learning. The consequence will be an undesirable difficulty effect.

## Future Directions for Research

Future research could show further boundary conditions for desirable difficulties. For example, for the testing effect, the degree of information given to students (e.g., allowing open book study, cf. Roelle and Berthold, 2017) could influence the differences between generating a solution and studying examples. Given that the main dependent variable in testing-effect studies is text comprehension and memory for text information, it would be interesting to further investigate whether the degree of element interactivity in the text materials moderates the testing effect. In this context, it should be noted that Hanham et al. (2017) conducted six experiments using textual material and found strong relations between the testing effect and the element interactivity of the information. Also regarding the testing effect, considering the research literature on elaborative memory strategies (e.g., Endres et al., 2017), it can be investigated if a test with elaborative prompts, in conditions of low element interactivity, is also productive for learning.

Concerning varied conditions of practice, future research could investigate whether the positive effects of practice variability would decrease or even reverse using high element interactivity information. Based on cognitive load theory, we can hypothesize that for high element interactivity information, practice variability may have negative rather than positive consequences. The high working memory load imposed by high element interactivity information may need to be compensated for by reducing variability. Another important direction for future research is related to investigating the role of element interactivity in other strategies that have been identified as desirable difficulties, such as distributed practice and interleaving practice (e.g., Rohrer and Pashler, 2010), although it should be noted that based on the current literature, there is little evidence that element interactivity plays a part in either the distributed practice or interleaving practice effects. Lastly, because the instructions that are used in desirable difficulty research could also be argued to be more challenging and engaging, future research could investigate the moderating effects of motivation on desirable difficulty effects.

## Conclusion

Some conflicting findings associated with desirable difficulties research possibly may be resolved by the concept of element interactivity within the framework of cognitive load theory. The experimental results on the testing effect and the generation effect have consistently shown that different results are obtained using high as opposed to low element interactivity information. The variability effect may similarly be dependent on element interactivity. We suggest that the element interactivity effect of cognitive load theory may provide a theoretical base indicating when difficulties are and are not desirable as well as providing a theoretical explanation for otherwise contradictory results.

## Author Contributions

OC drafted the manuscript. JC-A co-drafted the manuscript. FP and JS provided the critical revision of the manuscript. All the authors approved the final manuscript and were accountable for it.

## Conflict of Interest Statement

The authors declare that the research was conducted in the absence of any commercial or financial relationships that could be construed as a potential conflict of interest.
